# Supplementary material for: HIVprotI: an integrated web based platform for prediction and design of HIV proteins inhibitors
Source: J Cheminform. 2018 Mar 9;10:12. doi: 10.1186/s13321-018-0266-y (PMC5845081; doi:10.1186/s13321-018-0266-y)
Supplement: Supplementary file 2 — Additional file 2. Source code of HIVProtI web server. [file 13321_2018_266_MOESM2_ESM.zip › HIVprotI_Source-code/hivprotI/weka-3-6-6/doc/weka/clusterers/AbstractDensityBasedClusterer.html]

AbstractDensityBasedClusterer


---


|  |  |  |
| --- | --- | --- |
| **Overview** \| **Package** \| **Class** \| **Tree** \| **Deprecated** \| **Index** \| **Help** \| **Weka's home** | |  |
| **PREV CLASS**   **NEXT CLASS** | **FRAMES**    **NO FRAMES**     **All Classes** |  |
| SUMMARY: NESTED \| FIELD \| CONSTR \| METHOD | DETAIL: FIELD \| CONSTR \| METHOD |  |


---


## weka.clusterers Class AbstractDensityBasedClusterer

```
java.lang.Object
  weka.clusterers.AbstractClusterer
      weka.clusterers.AbstractDensityBasedClusterer
```

**All Implemented Interfaces:**: java.io.Serializable, java.lang.Cloneable, Clusterer, DensityBasedClusterer, CapabilitiesHandler, RevisionHandler

**Direct Known Subclasses:**: MakeDensityBasedClusterer, RandomizableDensityBasedClusterer

---

``` public abstract class AbstractDensityBasedClusterer extends AbstractClusterer implements DensityBasedClusterer ```

Abstract clustering model that produces (for each test instance)
an estimate of the membership in each cluster
(ie. a probability distribution).

**Version:**
:   $Revision: 1.1 $

**Author:**
:   Mark Hall (mhall@cs.waikato.ac.nz), Eibe Frank (eibe@cs.waikato.ac.nz)

**See Also:**: Serialized Form

---

**Constructor Summary**

`AbstractDensityBasedClusterer()`


| **Method Summary** | |
| --- | --- |
| `abstract  double[]` | `clusterPriors()`             Returns the prior probability of each cluster. |
| `double[]` | `distributionForInstance(Instance instance)`             Returns the cluster probability distribution for an instance. |
| `double` | `logDensityForInstance(Instance instance)`             Computes the density for a given instance. |
| `abstract  double[]` | `logDensityPerClusterForInstance(Instance instance)`             Computes the log of the conditional density (per cluster) for a given instance. |
| `double[]` | `logJointDensitiesForInstance(Instance inst)`             Returns the logs of the joint densities for a given instance. |
| `static DensityBasedClusterer[]` | `makeCopies(DensityBasedClusterer model, int num)`             Creates copies of the current clusterer. |

**Methods inherited from class weka.clusterers.AbstractClusterer**

`buildClusterer, clusterInstance, forName, getCapabilities, getRevision, makeCopies, makeCopy, numberOfClusters`

**Methods inherited from class java.lang.Object**

`equals, getClass, hashCode, notify, notifyAll, toString, wait, wait, wait`

**Methods inherited from interface weka.clusterers.Clusterer**

`buildClusterer, clusterInstance, getCapabilities, numberOfClusters`

**Constructor Detail**

### AbstractDensityBasedClusterer

```
public AbstractDensityBasedClusterer()
```


**Method Detail**

### clusterPriors

```
public abstract double[] clusterPriors()
                                throws java.lang.Exception
```

:   Returns the prior probability of each cluster.

    :   **Specified by:**: `clusterPriors` in interface `DensityBasedClusterer`
    :   **Returns:**: the prior probability for each cluster **Throws:**: `java.lang.Exception` - if priors could not be returned successfully

---


### logDensityPerClusterForInstance

```
public abstract double[] logDensityPerClusterForInstance(Instance instance)
                                                  throws java.lang.Exception
```

:   Computes the log of the conditional density (per cluster) for a given instance.

    :   **Specified by:**: `logDensityPerClusterForInstance` in interface `DensityBasedClusterer`
    :   **Parameters:**: `instance` - the instance to compute the density for **Returns:**: an array containing the estimated densities **Throws:**: `java.lang.Exception` - if the density could not be computed successfully

---


### logDensityForInstance

```
public double logDensityForInstance(Instance instance)
                             throws java.lang.Exception
```

:   Computes the density for a given instance.

    :   **Specified by:**: `logDensityForInstance` in interface `DensityBasedClusterer`
    :   **Parameters:**: `instance` - the instance to compute the density for **Returns:**: the density. **Throws:**: `java.lang.Exception` - if the density could not be computed successfully

---


### distributionForInstance

```
public double[] distributionForInstance(Instance instance)
                                 throws java.lang.Exception
```

:   Returns the cluster probability distribution for an instance.

    :   **Specified by:**: `distributionForInstance` in interface `Clusterer` **Overrides:**: `distributionForInstance` in class `AbstractClusterer`
    :   **Parameters:**: `instance` - the instance to be clustered **Returns:**: the probability distribution **Throws:**: `java.lang.Exception` - if computation fails

---


### logJointDensitiesForInstance

```
public double[] logJointDensitiesForInstance(Instance inst)
                                      throws java.lang.Exception
```

:   Returns the logs of the joint densities for a given instance.

    :   **Specified by:**: `logJointDensitiesForInstance` in interface `DensityBasedClusterer`
    :   **Parameters:**: `inst` - the instance **Returns:**: the array of values **Throws:**: `java.lang.Exception` - if values could not be computed

---


### makeCopies

```
public static DensityBasedClusterer[] makeCopies(DensityBasedClusterer model,
                                                 int num)
                                          throws java.lang.Exception
```

:   Creates copies of the current clusterer. Note that this method
    now uses Serialization to perform a deep copy, so the Clusterer
    object must be fully Serializable. Any currently built model will
    now be copied as well.

    :   **Parameters:**: `model` - an example clusterer to copy: `num` - the number of clusterer copies to create. **Returns:**: an array of clusterers. **Throws:**: `java.lang.Exception` - if an error occurs


---


|  |  |  |
| --- | --- | --- |
| **Overview** \| **Package** \| **Class** \| **Tree** \| **Deprecated** \| **Index** \| **Help** \| **Weka's home** | |  |
| **PREV CLASS**   **NEXT CLASS** | **FRAMES**    **NO FRAMES**     **All Classes** |  |
| SUMMARY: NESTED \| FIELD \| CONSTR \| METHOD | DETAIL: FIELD \| CONSTR \| METHOD |  |


---
